# Influence of meteorological conditions and climate on pollen season of the early-flowering woody taxa in Poland, Central Europe

**DOI:** 10.1007/s00484-025-02995-4

**Published:** 2025-09-02

**Authors:** Szymon Tomczyk, Małgorzata Werner, Małgorzata Malkiewicz, Beata Bosiacka, Łukasz Grewling, Agnieszka Grinn-Gofroń, Idalia Kasprzyk, Katarzyna Kluska, Barbara Majkowska-Wojciechowska, Dorota Myszkowska, Małgorzata Puc, Piotr Rapiejko, Monika Ziemianin

**Affiliations:** 1 Faculty of Earth and Environmental Sciences, University of Wrocław, Wrocław, Poland; 2 Institute of Biology, University of Szczecin, Szczecin, Poland; 3 Faculty of Biology, Department of Systematic and Environmental Botany, Laboratory of Aerobiology, Adam Mickiewicz University, Poznań, Poland; 4 Faculty of Biology and Nature Protection, University of Rzeszow, Rzeszów, Poland; 5 Department of Immunology and Allergy, Medical University of Łódź, Lódź, Poland; 6 Department of Clinical and Environmental Allergology, Jagiellonian University Medical College, Kraków, Poland; 7 Institute of Marine & Environmental Sciences, University of Szczecin, Szczecin, Poland; 8 Department of Otolaryngology with, Division of Cranio-Maxillo-Facial Surgery, Military Institute of Medicine, Warsaw, Poland; 9 Allergen Research Center Ltd, Warszawa, Poland

**Keywords:** Climate change, Pollen grains, Pollination, Early-flowering taxa

## Abstract

The aim of this study is to analyse the spatial and temporal variability of the pollen seasons in Poland and to demonstrate the impact of meteorological parameters on the course of the pollen seasons within the context of climate change over two decades 2003–2012 and 2013–2022. Five early-flowering plant taxa were selected for analysis: *Alnus* (alder), *Corylus* (hazel), *Populus* (poplar), *Ulmus* (elm), and *Salix* (willow). Daily airborne pollen data were collected using a Hirst-type pollen trap in six cities across Poland, representing different climatic conditions. Firstly, long-term air temperature trends were analysed to identify spatial and temporal climate variability. Secondly, the differences in pollen seasons between stations were compared to assess the spatial variation. Thirdly, pre-season meteorological influences on season onset and intensity were analysed. Finally, the influence of meteorological parameters on daily pollen grain concentration in the atmosphere was examined. The results show that, in the second decade, the strongest temperature increases occurred in early-year months, potentially influencing early-flowering taxa. Over a 20-year period, the period with the highest pollen grain concentration starts earlier and lasts longer for most stations and taxa. Meteorological influences on pollen dynamics shifted over time, leading to spatial, temporal, and species-specific variation across Poland. Air temperature, insolation time, and relative humidity notably influence daily pollen levels, especially for *Populus*, *Salix*, and *Ulmus* (correlation > 0.2), while *Corylus* and *Alnus* show weaker correlations (< 0.1). The analysis indicates a decline in correlation coefficient values over the study period, particularly for *Corylus* and *Alnus*.

**Supplementary Information:**

The online version contains supplementary material available at 10.1007/s00484-025-02995-4.

## Introduction

Anemophilous trees play a fundamental role in forest ecosystems, actively shaping their structure and dynamics. The wind pollination mechanism facilitates the efficient dispersal of pollen over long distances, contributing to the maintenance of genetic diversity while enhancing the adaptive potential of tree populations in response to changing environmental conditions. Beyond its primary function in reproduction, pollen serves as a valuable proxy for studying forest succession and evaluating the influence of anthropogenic factors on forest stability and resilience (Jetschni et al. 2023; Montiel et al. 2025).

In the context of air quality, there is a growing focus on the allergenic potential of pollen grains. This is particularly relevant for the pollen grains of anemophilous trees, which are produced and dispersed in substantial quantities. The human population is experiencing an increase in allergic rhinitis and allergic asthma, with the problem multiplying several times over the past decades (Dbouk et al. 2022; Kurganskiy et al. 2021; Lake et al. 2017; Ziska et al. 2019). It is estimated that between 10% and 25% of Europeans today, and possibly as many as 35%, especially in large cities, suffer from allergic problems caused by pollen grains. This makes allergies one of the major diseases of the 21 st century (Adamov and Pauling 2023; Annesi-Maesano et al. 2023; Buters et al. 2022). This problem is also relevant to Poland, where the prevalence of allergies is estimated to be on the rise, with approximately one-third of the Polish population suffering from allergic rhinitis (Krzych-Fałta et al. 2016; Lipiec et al. 2020; Samoliński et al. 2014). The intensity of allergic reactions is influenced by the concentration of pollen grains in the air, with variable symptom-triggering thresholds depending on pollen species, the affected population, and various environmental and individual factors (Rapiejko et al. 2007; Steckling-Muschack et al. 2021). In Poland, pollen grains from the Betulaceae family, particularly genera such as *Corylus* sp., *Alnus* sp., and *Betula* sp., are characterized by their significant allergic potential. The clinical relevance of other early-flowering tree genera, such as *Populus* sp., *Salix* sp., and *Ulmus* sp., is yet to be fully determined; however, these taxa are also considered allergenic (Grewling et al. 2023; Myszkowska and Majewska 2014; Weryszko-Chmielewska 2007).

Understanding the influence of meteorological conditions on pollen grain concentrations and the course of the pollen season is a key aspect of scientific research in this field (Dbouk et al. 2022; Lake et al. 2017; Malkiewicz et al. 2016). Several factors, in particular temperature and photoperiod, are critical for plant growth, development and pollen production. Precipitation and humidity substantially affect pollen release and dispersal, while wind speed is vital for the transport of pollen through the atmosphere (Laaidi 2001; Ravindra et al. 2022a, b; Werner et al. 2021). However, it should also be emphasized that weather conditions preceding the start of the pollen season are crucial for its progression, particularly those occurring within a few weeks of its onset (Grewling et al. 2012; Malkiewicz et al. 2016). In recent years, the influence of climate change on the pollen seasons has become more apparent. There is increasing evidence that the onset of the pollen season is occurring earlier, that the season is lasting longer, and that both the daily maximum concentration of pollen grains and the total number of pollen grains per season are increasing (Mousavi et al. 2022; Schmidt 2016; Ziska et al. 2019).

Although numerous studies have explored the relationship between climate change and pollen seasons, research in Central Europe remains limited, with a focus that is often short-term or territorially confined. Addressing this gap, our study takes a comprehensive approach by examining long-term data from multiple locations across Poland, with two main objectives. The first is to investigate the impact of climate change on the course and the intensity of pollen seasons of five common tree and shrub taxa in Poland. The second is to investigate whether the relationships between meteorological variables and pollen season features differ between two time periods, potentially reflecting long-term climatic trends. A key part of the study is the analysis of data from six stations representing different climatic conditions, ranging from the Baltic coastal area and lowlands to the highland foothills of the Tatra Mountains. The comparison between stations and consideration of long-term series data should allow for a comprehensive characterisation.

Therefore, this study tests the hypothesis that spatial differences in the dynamics of climate change are reflected in the variability of the pollen season. The premise of this article is to focus on the early flowering tree genera, i.e. *Corylus* sp. (hazel), *Alnus* sp. (alder), *Populus* sp. (poplar), *Salix* sp. (willow), and *Ulmus* sp. (elm), which release pollen during the first months of the year. The turn of winter and spring is characterised by high variability in meteorological conditions and hence strongly influenced by climate change (Błaś and Ojrzyńska 2024; Kundzewicz and Matczak 2012). In addition, the pollen grains of the selected taxa have allergenic properties while the distribution of these trees is widespread across Central Europe, emphasising the importance of analysing the impact of climate change on the health and well-being of the European population (Johnson and More 2006; Senata et al. 2021).

## Materials and methods

### Study area

**Fig. 1:**
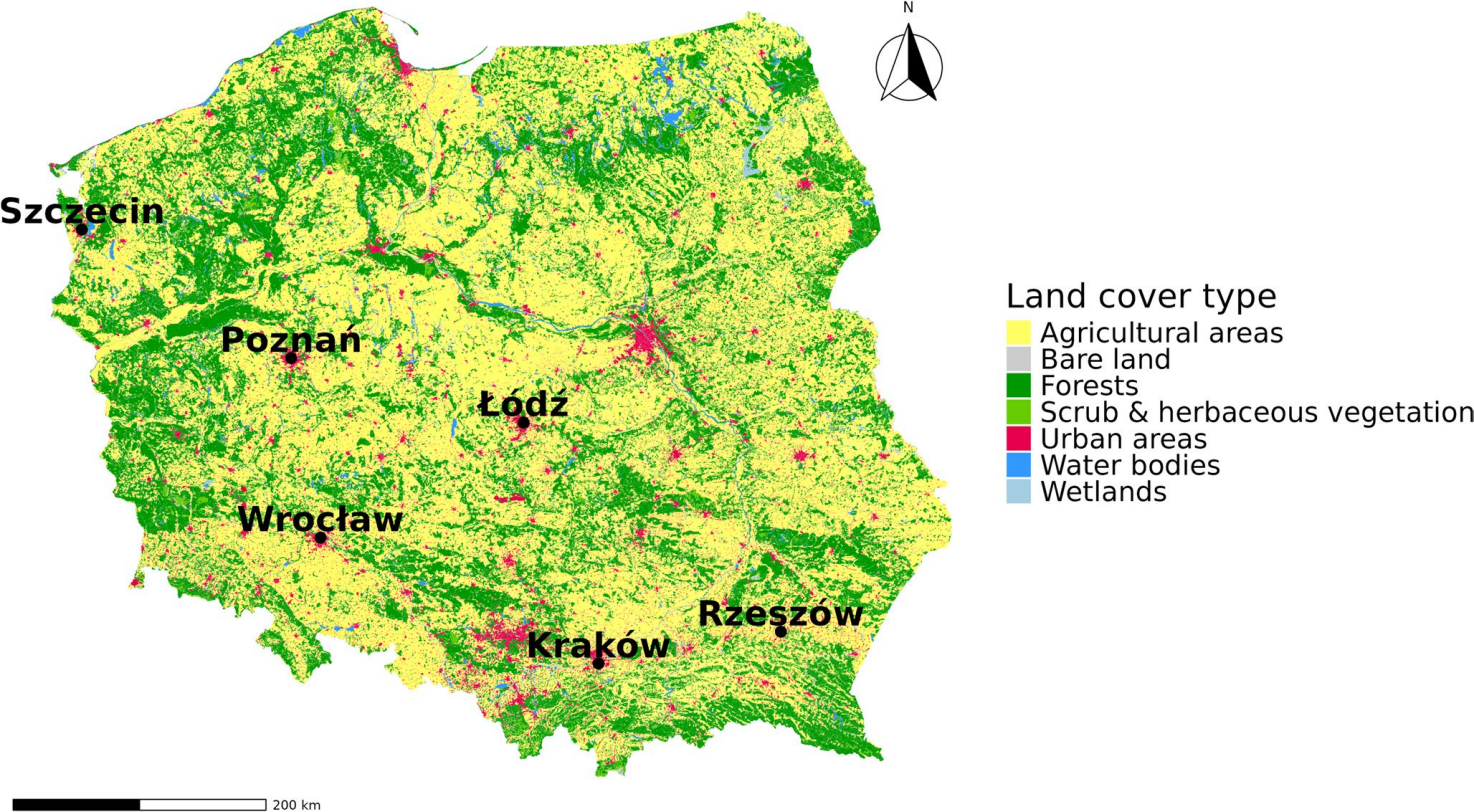
Land cover types in Poland based on CORINE Land Cover 2018, with 100-meter resolution

Data were collected from six stations in Poland, namely Szczecin, Poznań, Wrocław, Kraków, Rzeszów, and Łódź (Fig. 1). This selection allows for a comparison of stations located in different geographical areas with different climatic conditions. Some of the selected stations are quite distant from each other, covering an area of approximately 500 km from south to north and 400 km from west to east, thereby allowing for a wider range of analysis. According to Koppen’s climate classification, stations located in western and south-western Poland are characterised by a more oceanic climate - Cfb, while others located further east are in the warm-summer humid continental climate zone - Dfb (Kożuchowski 2011).

The climatic conditions of the analysed stations are shown in Table 1. Poland’s climate is temperate and transitional, with the strongest relevance due to air masses such as maritime (direction W), but also polar (direction N), continental (direction E), and subtropical (direction S) (Błaś and Ojrzyńska 2024; Kundzewicz and Matczak 2012). The highest annual mean air temperature is recorded at stations located in the west and south-west of Poland (9 to 10 °C). The average air temperature decreases towards the east and north due to the fact that this area tends to be dominated by colder air masses in winter. The highest precipitation values are observed in southern Poland (550–650 mm). The stations in the central part of the country have the lowest annual precipitation totals (Błaś and Ojrzyńska 2024; Kożuchowski 2011).

**Table 1:** Location of monitoring stations and mean climatic conditions (2003–2022)

Station	Climate	Station	Climate
**Wrocław** 51.12 N, 17.03 E	10,68 °C 549,4 mm	**Szczecin** 53.44 N, 14.55 E	10,28 °C 548,6 mm
**Poznań** 52.41 N, 16.89 E	10,12 °C 551,0 mm	**Rzeszów** 50.03 N, 22.01 E	9,13 °C 641,6 mm
**Łódź** 51.77 N, 19.48 E	8,99 °C 574,9 mm	**Kraków** 50.06 N, 19.96 E	9,03 °C 692,2 mm

Regarding the surroundings of the pollen monitoring stations, the analysed trees do not occur in larger quantities near the stations studied (Table 1 S). However, it is worth mentioning the proximity of the stations in Szczecin, Poznań, and Łódź to larger green areas, such as parks, and the proximity of the station in Kraków to the nearby Botanical Garden. Wrocław and Rzeszów stations are typical city-centre stations. The land cover around the six monitoring stations in Poland varies notably. Urban areas dominate the surroundings of Łódź, Kraków, and Wrocław, while Rzeszów, Poznań, and Szczecin are more strongly surrounded by a mix of agricultural land and forested areas, indicating different exposure to vegetation types and potential allergen sources (Fig. 1).

### Aerobiological data

Aerobiological data cover the period 2003–2022 and were collected using the Hirst-type volumetric pollen trap (Hirst 1952). The microscopic slides were scanned along four horizontal transects and the results were expressed as daily mean pollen concentration according to the formula recommended by the International Association for Aerobiology (Galán et al. 2014). The pollen season was determined using the 95% method (Nilsson and Persson 1981). The start of the pollen season was the day when the cumulative sum of pollen grains exceeded 2.5% of the total sum, and the end was when the cumulative sum exceeded 97.5% of the total sum. The pollen season characteristics also include Seasonal Pollen Integral (SPIn) - the total amount of pollen for the whole season (Jato et al. 2006; Rodríguez-Rajo et al. 2010). The analysis focused on the following tree taxa: *Corylus*, *Alnus*, *Populus*,* Salix*, and *Ulmus*.

### Meteorological parameters

To begin with, we examined long-term trends in air temperature by analysing monthly mean values from 2003 to 2022. We then analysed a set of meteorological parameters, which were divided into two main groups. The first group includes three key variables - mean air temperature (t2m), sunshine duration (ins), and daily precipitation (rr) - selected for in-depth analysis of their impact on pollen season characteristics. These variables are among the most commonly studied in aerobiological research and have well-established roles in determining the timing and intensity of pollen seasons. Unlike daily pollen concentrations, which can be affected by a wide range of short-term meteorological variations, seasonal parameters tend to reflect cumulative or trend-related influences over longer periods.” (Kubik-Komar et al. 2018; Malkiewicz et al. 2016). For this reason, the second group comprises a broader selection of variables considered particularly relevant to daily pollen concentrations. This group includes: mean daily temperature (t2m, °C), maximum daily temperature (tmax, °C), minimum daily temperature (tmin, °C), minimum soil temperature at a depth of 5 cm (tmin soil, °C), sunshine duration (ins, hours), mean relative humidity (rh, %), daily precipitation (rr, mm), mean wind speed (ws, m/s), and sea level pressure (slp, hPa). The temporal range of the selected meteorological variables corresponds to the duration of the pollen season for each taxon and year, as defined by the 95% method. All data were sourced from the meteorological stations of the Institute of Meteorology and Water Management located closest to the respective aerobiological monitoring sites (Czernecki et al. 2020).

### Statistical analysis

The trend of increasing air temperature was analysed using the Mann–Kendall test, accompanied by the p-value and Kendall’s rank correlation coefficient, applied separately to two consecutive decades: 2003–2012 and 2013–2022. This preliminary step was crucial for establishing the climatic background relevant to the aerobiological analysis, justifying the division of the study period into two distinct sub-periods, and confirming that the focus was on early-flowering species. Air temperature was chosen as the primary variable because it is one of the most direct and sensitive indicators of climate change, whereas other meteorological parameters often reflect indirect or secondary effects (Twardosz et al. 2021).

The standard pollen calendars were prepared to present the course of pollen seasons across different stations and taxa. Calculations were based on daily average pollen concentrations for each pollen type. For each year separately, the daily average values were summed up to obtain the annual total pollen count. Based on this cumulative pollen sum, three seasonal intervals were defined and presented on the calendars using different colours. The main pollination period (red) represents 80% of the annual total pollen grains; it begins when 10% of the cumulative annual value is reached and ends when 90% is reached, indicating the period with the highest pollen levels. The early and late pollination period (orange) covers 90% of the total annual pollen, starting when 0.5% is reached and ending when 99.5% is reached, showing a broader season around the main period. The possible occurrence period (yellow) represents 100% of the annual pollen and is defined as the entire period between the first and last day when any pollen grains were detected. The ranges of the classes (red, orange and yellow) are marked on the plot for individual days rather than being aggregated into weeks or other periods. Finally, the annual calendars are averaged over the analysed periods (2003–2012, 2013–2022) and presented in the manuscript. In addition, to assess changes in the intensity of seasonal pollen production (SPIn) between two decades (2003–2012 and 2013–2022), a one-way analysis of variance (ANOVA) was performed separately for each taxon. Tukey’s Honest Significant Difference (HSD) test was applied as a post-hoc procedure to identify statistically significant differences between groups. The results were visualized using boxplots with significance levels indicated.

The Spearman correlation was used to identify the correlations between meteorological parameters (t2m, ins, and rr), calculated as monthly averages preceding the pollen season, and two key pollen season characteristics: the start date of the season and the SPIn. For *Corylus*, meteorological data from December and January were considered, for *Alnus*, January and February, and for *Populus*,* Salix*, and *Ulmus*, February and March. This analysis was carried out separately for each station, dividing the period analysed into two decades (2003–2012 and 2013–2022) in order to show changes in the strength of the correlations over time.

Finally, we analysed the correlations between meteorological parameters and pollen grains concentration. For analysis selected all variables mentioned in 2.3. section due to fact that amount of pollen in air is determined by many factors that’s why it is crucial to examine all. In order to minimize the risk of spurious significance in statistical tests, we applied the Bonferroni correction. All calculations were performed in the R programming environment (RStudio Team 2025).

## Results

### Temporal and Spatial patterns of air temperature increase

**Fig. 2:**
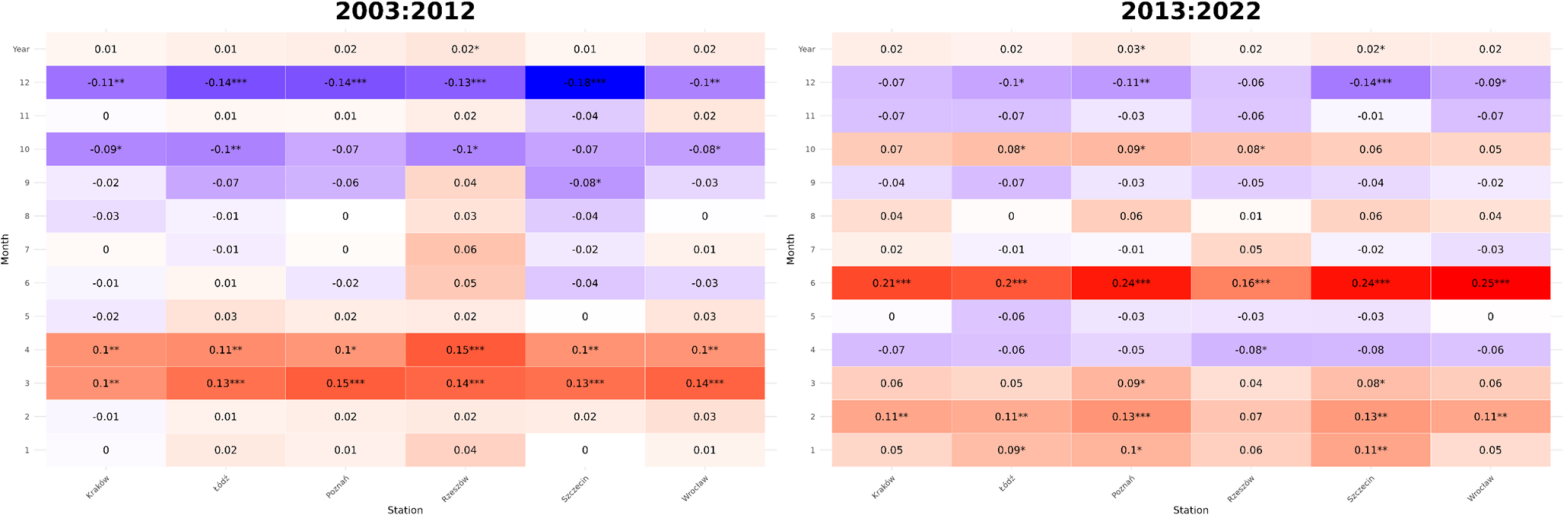
Trend of mean air temperature increase from 2003 to 2022, divided by months and measurement stations (statistically significant results marked as * for *p* < 0.05, ** for *p* < 0.01, and *** for *p* < 0.001). The X-axis represents the years (2003–2012 and 2013–2022), and the Y-axis shows the Kendall’s tau trend for each month, calculated based on the temperature values for each decade

In the first decade (Fig. 2), the most significant temperature increases were observed in March and April (correlation coefficient ranging from 0.1 to 0.15), whereas for the other periods, the changes were statistically insignificant, with even a decline noted towards the end of the year. However, in the second decade (Fig. 2), a greater number of months exhibited statistically significant temperature increases, particularly in June (correlation coefficient ranging from 0.16 to 0.25) and early months of the year, with a pronounced rise in January and February (correlation coefficient ranging from 0.05 to 0.13). This trend was especially evident among stations in western and central Poland, further supporting the validity of the study objectives and research hypothesis.

### Pollen season characteristics and their Climatic determinants: timing, intensity, and variability

**Fig. 3:**
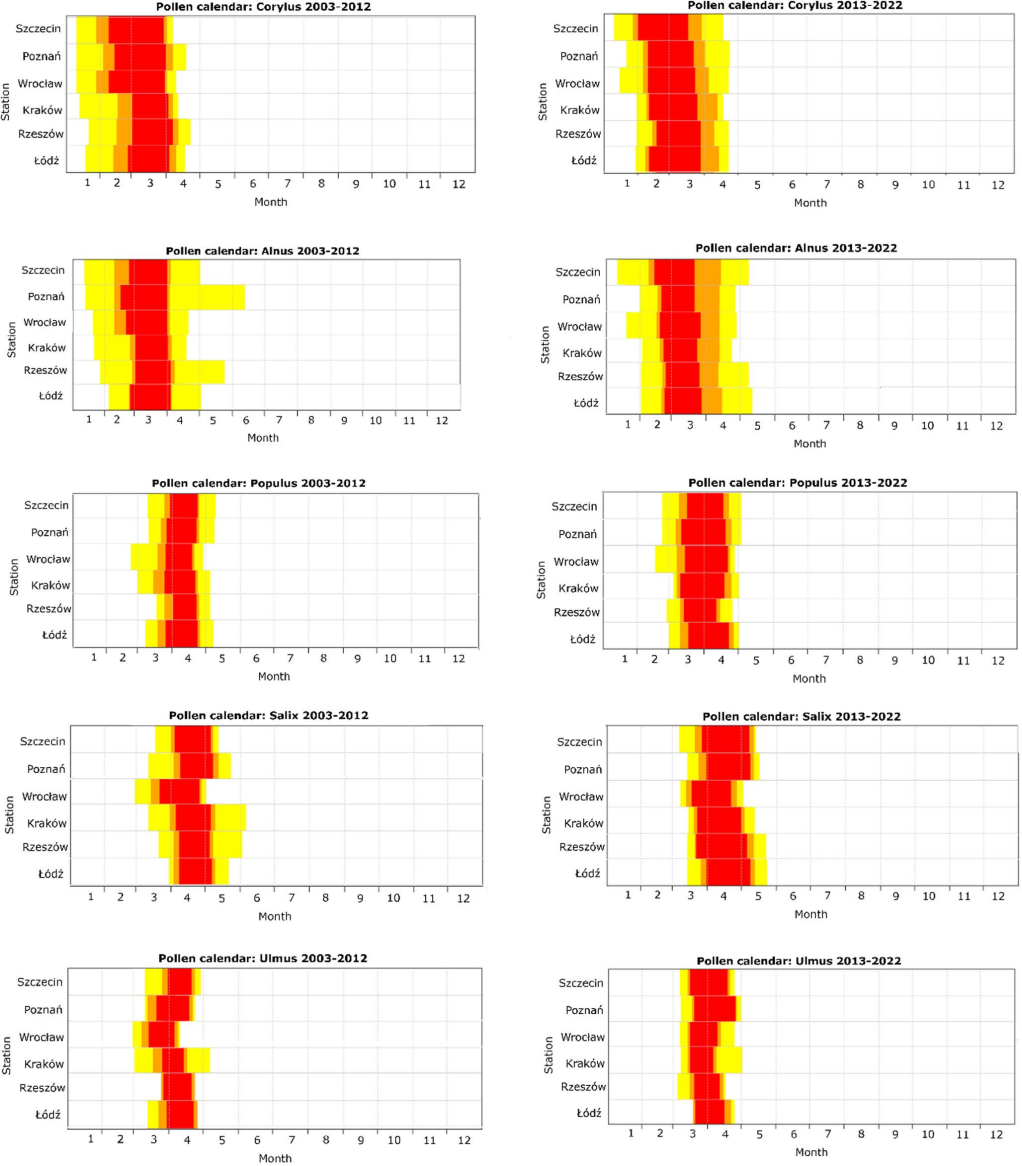
Pollen calendars for taxa by station divided into two periods: 2003–2012 and 2013–2022 (yellow − 100% of pollen grains for the season, orange − 90% of pollen grains for the season, red − 80% of pollen grains for the season)

Pollen season dynamics of *Corylus* and *Alnus* showed remarkable differences over studied period (Fig. 3). The onset of the pollen season was clearly at a later time in the second decade (2013–2022) compared to the first decade (2003–2012), resulting in a shorter overall duration for the season. In contrast, the most intense part of the season (red colour) tends to occur earlier. Similar trends have been observed for other taxa, though less pronounced. In addition, the most intense part of the season lasted longer in the second decade (2013–2022), especially for *Corylus*, *Populus*, and *Salix* (Fig. 3).

Comparing the results of the first and second decades, the differences between the stations were smaller in the second decade. The most intense part of the season tended to start earlier, whilst the dynamics of the pollen season became more uniform across stations. In stations such as Wrocław, Szczecin, Poznań, and Kraków, the pollen season started earlier for most of the studied taxa (Fig. 3). Conversely, in Łódź and Rzeszów, the pollen seasons tended to start later compared to the other stations. There were also differences in seasonal dynamics between stations. In Rzeszów and Łódź, the pollen season began without a transitional phase, indicating a rapid onset of the main season. In contrast, the other stations showed a prolonged period of low pollen grain levels, leading to a longer overall season (Fig. 3).

**Fig. 4:**
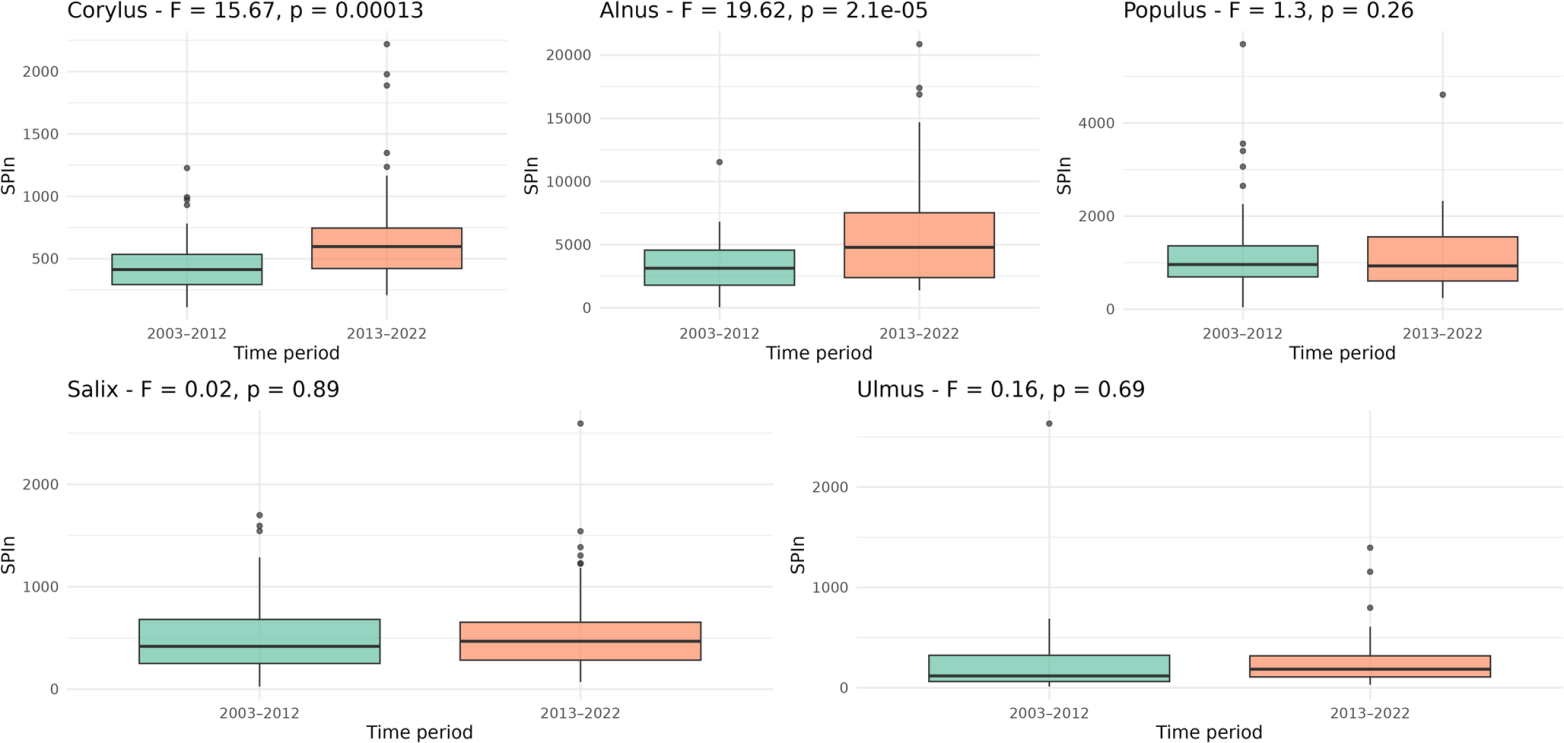
Boxplots of Seasonal Pollen Index (SPIn) for five tree taxa (*Corylus*, *Alnus*, *Populus*, *Salix*, *Ulmus*) in two decades (2003–2012 and 2013–2022). The plots present the results of one-way ANOVA, with F-values and *p*-values.

In the ANOVA analysis of the seasonal pollen sum (SPIn) between two decades — 2003–2012 and 2013–2022 (Fig. 4) — clearly differentiated trends were observed depending on the tree taxon. Among the five taxa analysed, only two — *Corylus* and *Alnus* — showed a statistically significant increase in SPIn during the most recent decade. For *Corylus*, the F-statistic was 15.67 with a p-value of 0.00013, while for *Alnus*, the F-statistic reached 19.62 with *p* = 2.1e-05. In both cases, the mean SPIn values were significantly higher in 2013–2022 compared to 2003–2012, indicating an intensification of pollen production in recent years. Although the boxplots illustrate the medians and spread of the data, the ANOVA results confirm that the differences in means between decades were statistically significant. For the remaining genera — *Populus* (F = 1.3, *p* = 0.26), *Salix* (F = 0.02, *p* = 0.89), and *Ulmus* (F = 0.16, *p* = 0.69) — the differences between decades were not statistically significant.

**Fig. 5:**
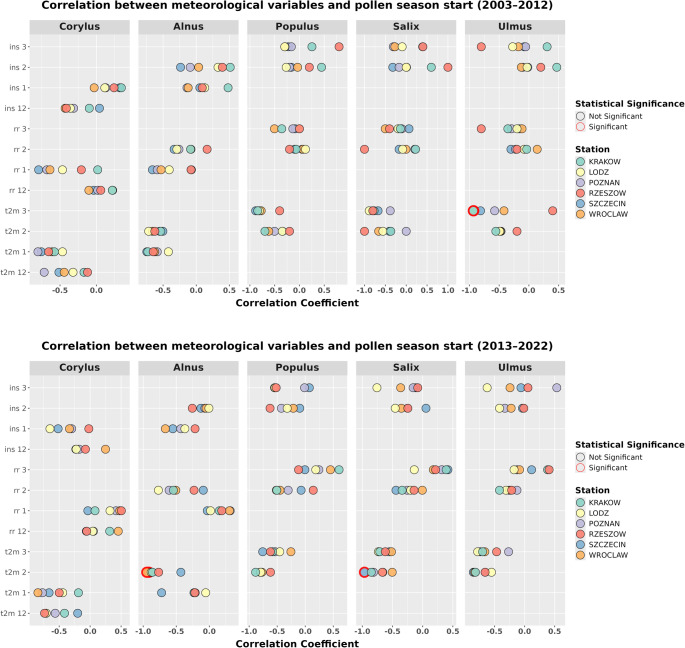
Significance of the effect of variables on pollen season start date 2003–2012 and 2013–2022, calculated using the 95 method (statistically significant marked as * for *p* < 0.05, ** for *p* < 0.01, and *** for *p* < 0.001). Each variable is labeled with the corresponding month number, while the x-axis varies according to the taxon under study

Meteorological conditions preceding the onset of the pollen season significantly correlated to starting date of pollen occurrence in the air for the studied plant taxa (Fig. 5). The observed negative values indicated an earlier onset of the pollen season, particularly in relation to the influence of air temperature. For *Corylus*, t2m in January showed a correlation coefficient below − 0.5 with the start of the season at most stations during the 2003–2012 period. However, in the 2013–2022 period, higher correlation values between t2m and the start of the season were observed for December. In the case of *Alnus*, a decrease in the correlation coefficient between t2m and the start of the season was observed in January. In February, the correlation strengthened (*r* < −0.7) at all stations except Szczecin, which showed the highest value among all stations in January. For other taxa, a clear temporal shift in the highest correlation coefficient values was observed, moving from March to February (*r* < −0.5); for *Salix* in Szczecin, this relationship was statistically significant. It should be emphasized that identifying a clear trend in the correlation values between t2m and the season onset across different stations was not explicitly evident.

No station exhibits statistically significant correlations between precipitation and the start of the season for the analysed taxa (Fig. 5). The highest negative correlation between rr in December and the start of the pollen season was recorded during the 2003–2012 period at multiple stations (Szczecin, Poznań, and Wrocław). However, in the second period (2013–2022), the correlation between rr and the start of the season shifted toward a positive value. For *Alnus*, an increase in correlation coefficient between values of the first and second period was observed in February, indicating a shift towards an inverse relationship, whereas in January, most values became positive. For other taxa the strengthening of negative correlation values was noted in February, with a subsequent shift to positive values in March. No distinct spatial trends were identified.

A reverse relationship was observed between insolation time and the start of the season compared to the two previously analysed parameters (Fig. 5). In the case of *Corylus*, the correlation’s value between the first and second period increased for January (Łódź and Szczecin; correlation coefficient < −0.5), while for December, correlation values declined. For *Alnus*, negative correlation values increased in January, particularly for Wrocław (correlation coefficient < −0.55). At other stations, a slight increase in negative correlation values was observed over time, although these were not statistically significant and did not exhibit clear spatial patterns.

For most stations, air temperature preceding the pollen season during the first analysed period (2003–2012) exhibited a negative correlation with SPIn recorded during the season (Fig. 1S), particularly for *Corylus* and *Alnus*. In the case of *Corylus*, this relationship was strong in December for Wrocław and Szczecin (correlation coefficient < −0.65). Other taxa generally showed either no correlation or positive relationships. In the second period (2013–2022), a shift towards a positive correlation between the air temperature preceding the pollen season and SPIn was observed for most stations and taxa, particularly for *Ulmus* in both analysed months and for *Salix* in March (correlation coefficient > 0.2). No clear trends in correlation values over space were observed. However, higher correlation values for most taxa in both periods were consistently recorded at the two southern stations, Kraków and Rzeszów.

Regarding precipitation, in the first period (2003–2012), *Corylus*,* Alnus*, and *Ulmus* exhibited a negative correlation with SPIn, whereas *Populus* and *Salix* showed a positive correlation (Fig. 1S), though not statistically significant for all. However, in the 2013–2022 period, an inversion of these relationships was observed. The recorded values did not indicate clear spatial patterns in correlation coefficient changes.

In the first period, the insolation time had a positive effect on seasonal pollen abundance, particularly for *Ulmus* in both analysed months and *Alnus* in February, although these relationships were not statistically significant. Other taxa exhibited varying values depending on the month and station. Over time, most stations recorded negative correlation values, particularly for *Populus* and some stations in the case of *Ulmus and Salix* (correlation coefficient < −0.4). During the 2013–2022 period, Rzeszów and Wrocław (Fig. 1S) consistently showed the highest correlation coefficient values between ins and SPIn.

### Influence of meteorological parameters on daily pollen grain concentration

Thermal variables, especially maximum and daily mean temperatures, showed a positive correlation with pollen grain concentration (Figs. 2, 3, 4, 5 and 6, 7 S). The highest values were observed for *Salix* (*r* ranging from 0.2 to 0.3 on average), followed by *Populus* and *Ulmus* (*r* between 0.1 and 0.2 on average). The correlation strength was comparable for all analysed stations, with only Wrocław (Fig. 5S) showing a slightly lower correlation. No significant temporal changes in the correlation coefficients were observed, except for a slight decrease in Kraków (Fig. 3S). The pattern was similar for the daily air minimum temperature and minimum soil temperature, although the correlation strength was weaker. For *Corylus* and *Alnus*, the correlation coefficients for thermal variables were clearly lower than for other taxa, and for the most part a negative relationship was observed over time. The highest correlation values between relative humidity and pollen concentration were found in three plant taxa: *Salix*, *Ulmus*, and *Populus* (*r* ranging from − 0.4 to −0.1). For *Corylus* and *Alnus*, the correlation coefficients were lower and often not statistically significant.

When comparing the two decades, there were no noticeable temporal differences. Insolation time showed high correlation values with pollen grains. This was particularly evident for *Populus*, *Salix*, and *Ulmus* (with *r* ranging from 0.1 to 0.3 on average), especially at the Szczecin, Poznań, and Łódź stations (Figs. 2 S, 4 S, and 6 S). Temporal changes were observed at two stations, Poznań and Rzeszów (Figs. 4 S and 7 S), where the correlation values decreased and were even negative in some cases.

The analysis showed that daily precipitation, daily mean sea-level pressure, and daily mean wind speed exhibited lower correlation coefficients with pollen grain concentrations. However, precipitation, particularly for *Populus*,* Salix*, and *Ulmus*, showed statistically negative significant results. For *Corylus* and *Alnus*, it was noticeable that the correlation between concentrations and wind speed reached statistically significant values at some stations. Nevertheless, no spatial differences in these values were observed.

## Discussion

Recent research suggests that climate change will lead to longer pollen seasons and increased pollen production by plants, potentially altering the ecosystem dynamics and plant reproductive patterns (Ziska et al. 2019). In addition, the variability of the pollen season is increasing. This is reported especially for early-flowering plant taxa, as confirmed by numerous studies in Europe and Poland (Dbouk et al. 2022; Lake et al. 2017; Malkiewicz et al. 2016). Our analysis of two consecutive decades ending in 2022 indicates an increase in the amount of pollen produced at six monitoring stations in Central Europe. However, it should be noted that this trend was particularly pronounced for the earliest pollinating plants, namely *Corylus* and *Alnus*. This aspect also highlights the considerable annual variability in meteorological conditions, as demonstrated in the study by Malkiewicz et al. (2016). Numerous studies suggest that rising CO₂ concentrations may lead to increased pollen production (Montiel et al. 2025). Also emphasized is that air pollution in large cities, such as those where the monitoring stations were located, may even contribute to a reduction in pollen grain abundance (Jetschni et al. 2023). Additionally, the potential influence of changes in tree population density near the stations should be considered, although this factor is unlikely to have markedly affected the time period under study.

A comparison of two decades (2003–2012 and 2013–2022) showed that the first pollen grains tend to appear later. However, the period of higher pollen concentration in the air for most of the analysed taxa now starts earlier and lasts longer, which is particularly important for allergy sufferers. Previous studies in Poland have emphasised that the pollen season of early flowering plant taxa is extremely dynamic. Winter conditions, characterised by considerable variability, are causing differences in the start of the season that can reach up to two months (Malkiewicz et al. 2016). Characteristics of the pollen season also change significantly from one year to another (Dbouk et al. 2022; Lake et al. 2017). For other taxa (e.g. Poaceae and *Betula*), more pronounced trends are observed, as highlighted in studies covering both Poland and other European countries (Adams-Groom et al. 2022; Puc et al. 2015; Kubik-Komar et al. 2018; Ziska et al. 2019). However, it should be noted that some studies focusing on Western and Central Europe suggest a significant trend towards an earlier start of the pollen season for early-flowering plant taxa, as indicated by this work (Picornell et al. 2023; De Weger et al. 2021).

Airborne pollen monitoring is a critical tool for investigating the reproductive phenology of anemophilous plants, serving as an effective bioindicator in regions with fluctuating pollen concentrations. Pollen production and dispersal are influenced by a range of factors, including plant genotype, age, size, phenology, and environmental conditions (Ruiz-Valenzuela and Aguilera, 2018). Our study underscores the increasing importance of pollen season dynamics, particularly for early-flowering taxa. Research suggests that climate change is profoundly altering the timing and intensity of pollen seasons, potentially affecting plant behavior, genetic diversity, and the adaptive capacity of tree populations in response to evolving climatic and anthropogenic pressures (López-Orozco et al. 2023).

A comparison between stations showed that climatic conditions had a pivotal role in shaping the seasons. The importance of the station’s geographical location was also demonstrated in the study by Myszkowska et al. (2010) and Puc and Kasprzyk (2013). Stations located in more favourable climatic conditions (western and southern Poland) showed more intense and longer pollen seasons. However, the analysis revealed that the trend of rising air temperatures was most pronounced at stations in central and western Poland between 2013 and 2022. These findings point to the necessity of examining additional meteorological variables influenced by climate change.

The analysis of the significance of variables by matrix correlation showed that, in agreement with previous studies, thermal parameters are particularly important in shaping pollen grain concentration (Novara et al. 2016; Piotrowska-Weryszko 2013; Schramm et al. 2021). This study also showed that insolation time, highlighted in many studies as a key factor for pollen release (Puc and Kasprzyk 2013; Bilińska et al. 2019), has a high positive correlation with daily concentration levels. In the context of pollen release and dispersal, the occurrence of high relative humidity is significant for washing out pollen grains and inhibiting their release from inflorescences (Ravindra et al. 2022a, b; Grewling et al. 2023), which was also demonstrated in this study. Atmospheric precipitation was shown to have less influence on pollen concentration than relative humidity.

Our study also identified differences in correlations’ strength between pollen concentrations and meteorological variables across different taxa. It is frequently emphasized in the literature that early-flowering taxa (*Corylus* and *Alnus*) exhibit distinct characteristics compared to later-flowering tree species (Malkiewicz et al. 2016; Nowosad et al. 2018). The results indicate that the analysed taxa can be categorized into two groups: (1) *Corylus* and *Alnus*; and (2) *Populus*, *Salix*, and *Ulmus*. In the case of the first group, statistically significant correlations between the meteorological variables and pollen concentrations were difficult to identify. The high variability of meteorological conditions at the beginning of the year likely contributed to the lack of correlation (Malkiewicz et al. 2016; Puc and Kasprzyk 2013). For the remaining taxa, correlation coefficients tended to be higher and were statistically significant for most meteorological parameters.

Over time, a decline in correlation strength and a shift in correlation patterns have been observed, particularly for *Corylus* and *Alnus*. Climate change may have contributed to increased weather variability and more abrupt day-to-day fluctuations. This variability makes it challenging to obtain strong correlations between meteorological parameters and pollen concentrations, which could become a major issue in the near future, particularly for modelling and forecasting pollen concentrations (Ren et al. 2022; Ziska et al. 2019). Wrocław did not exhibit a significant upward trend in temperature between 2013 and 2022, in contrast to the other stations. However, it is important to note that Wrocław stands out as having the most favourable climatic conditions for vegetation growth among all the stations studied. Yet even minor shifts in these climatic factors could potentially disrupt established correlations between pollen concentrations and meteorological parameters.

The analysis of the significance of meteorological variables (*t2m*, *ins*, and *rr*) in shaping pollen season’s characteristics (starting date and seasonal pollen integral, SPIn) confirmed previously documented scientific findings. Meteorological conditions, one to two months before the start of the pollen season, can influence its progression, particularly the season’s onset, as plants require favourable conditions for development and flowering (Grewling et al. 2012; Malkiewicz et al. 2016). The results indicate that air temperature plays a crucial role in determining the start of the pollen season. However, insolation time also exhibits statistically significant correlations with the season’s onset, as does, to some extent, cumulative precipitation.

No statistically significant relationships were found between pre-season meteorological conditions and total pollen production for the analysed parameters. Furthermore, no clear spatial trends were observed for the pollen season’s characteristics either, suggesting a lack of consistent spatial patterns in the strength and nature of these correlations. That being said, temporal changes were evident.

In most cases, the month with the highest correlation coefficients between the meteorological variables and the start of the season shifted to an earlier point in the season, accompanied by changes in the strength and direction of the correlations over time. During the 2013–2022 period, most stations exhibited a lack of correlation, indicating that climate change may have complicated the identification of key meteorological drivers shaping the pollen season. In the future, climate change may require researchers to better identify those meteorological variables and pre-season time periods that are most relevant for modelling and forecasting pollen season dynamics (Ziska et al. 2019).

## Summary and conclusions

This study examined early-flowering plant taxa - *Corylus*, *Alnus*, *Populus*, *Salix*, and *Ulmus* - across six stations in Poland, focusing on changes in pollen seasons over two decades (2003–2022). Key findings include:

1. Over the 20-year period, the first appearance of pollen grains occurred later, while the peak pollen period started earlier and lasted longer in the second decade.
2. Seasonal Pollen Integral (SPIn) values increased for all taxa, particularly for *Alnus* and *Corylus*, in southeastern Poland.
3. Thermal variables, insolation, and relative humidity were the main meteorological factors influencing daily pollen concentrations.
4. Later-flowering trees (*Populus*, *Salix*, *Ulmus*) had stronger correlations with meteorological variables over time, while *Corylus* and *Alnus* showed weaker correlations.
5. Temperature and insolation prior to the pollen season were key determinants of its onset, with their influence progressively shifting to earlier periods over the 20 years, especially for *Corylus* and *Alnus*.

These findings confirm that climate change affects flowering phenology in diverse ways, with regional variations in pollen season trends. However, no clear spatial patterns emerged regarding the influence of meteorological factors. The results suggest that climate change could exacerbate allergic risks in Poland, complicating the timing of antihistamine use. Additionally, shifts in pollen season dynamics and meteorological conditions could impact forest ecosystems, altering pollen dispersal, genetic diversity, and resilience to climate change. These trends may also extend to Central Europe, as indicated by the consistency across multiple monitoring stations.

## Electronic supplementary material

Below is the link to the electronic supplementary material.

ESM 1

## Data Availability

The data are available from the corresponding author upon reasonable request.
