# Supplementary material for: Influence of meteorological conditions and climate on pollen season of the early-flowering woody taxa in Poland, Central Europe
Source: Int J Biometeorol. 2025 Sep 2;69(10):2781–93. doi: 10.1007/s00484-025-02995-4 (PMC12540614; doi:10.1007/s00484-025-02995-4)
Supplement: Supplementary file 1 — ESM 1 [file 484_2025_2995_MOESM1_ESM.docx]

Table 1S. Characteristics of the station's surrounding

| **Station** | **Land cover** | **Station** | **Land cover** |
| --- | --- | --- | --- |
| **Wrocław**    51.12 N, 17.03 E | The sampler is placed in the city centre, from the south, the building is surrounded by an alley of plane trees, while several horse-chestnut trees. Small birches grow to the north of the building. | **Szczecin**    53.44 N,  14.55 E | The measuring site is located in Szczecin Śródmieście district in direct vicinity 0.5 km NW from the Jan Kasprowicz Park (many different species of plants, which mainly include pine, plane, birch, linden, beech and oak). |
| **Poznań**    52.41 N,  16.89 E | The station is located in downtown area of Poznań. About 500 meters to the south there is a small park with oaks, and to the west, a street lined with plane trees. The building is surrounded by many young birches. | **Rzeszów**    50.03 N,  22.01 E | The sampler is located at 12 m a.g.l. in the city centre. There are University campus buildings and big crossroads nearby. About 300 m to the west there is river with green spaces and to the south, east and north there are blocks of flats estates. Around the station lime and red oaks trees dominate. In street and estate greenery also trees of silver birch, poplars, maples and spruces are common. |
| **Łódź**    51.77 N,  19.48 E | The station is located in city centre, at the distance of about 30 km from the geometric centre of Poland. In the immediate vicinity, birch trees grow along with a few Lombardy poplars, sugar maples, ginkgo, ashes, and ornamental apple trees. There is a small park nearby, named after S. Staszic, where numerous species of trees and bushes are grown, both national and foreign. | **Kraków**    50.06 N,  19.96 E | The station is located near the Botanical Garden in the city center. The area features dense buildings with a green belt, where horse chestnuts, lindens, maples, poplars, and other ornamental trees and shrubs are prevalent. |


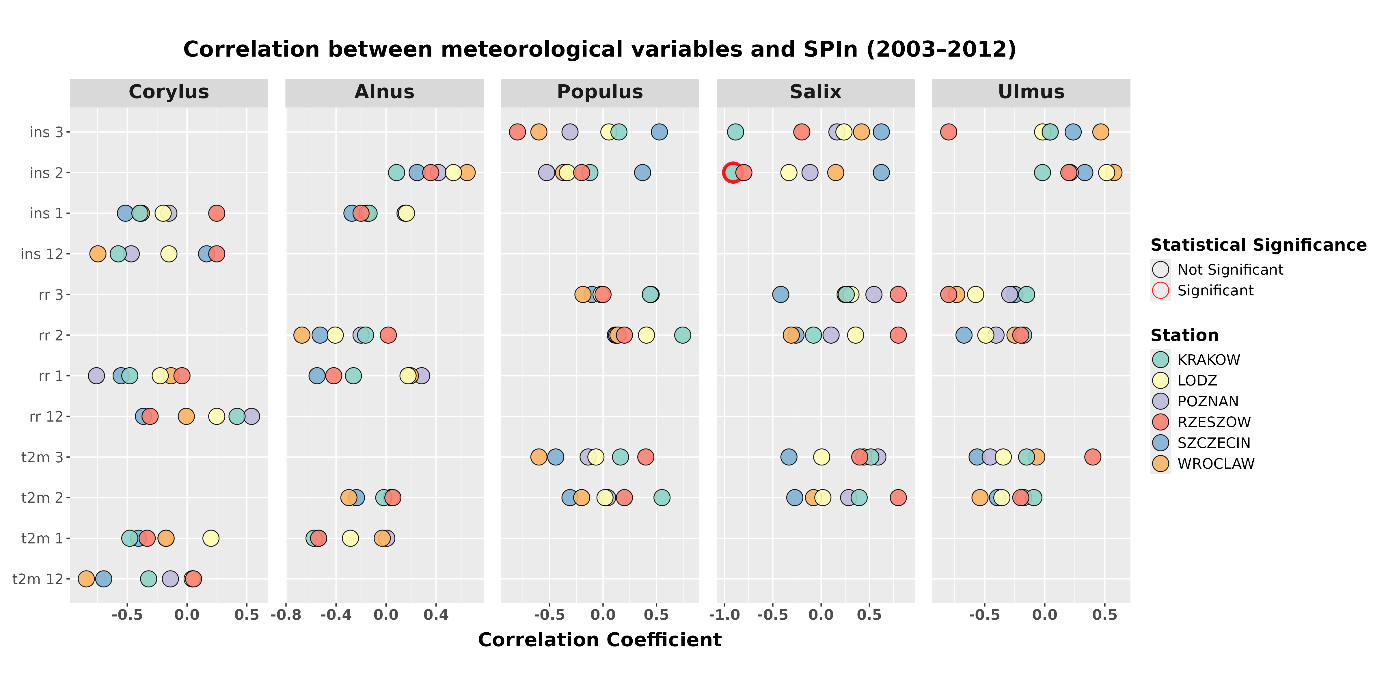


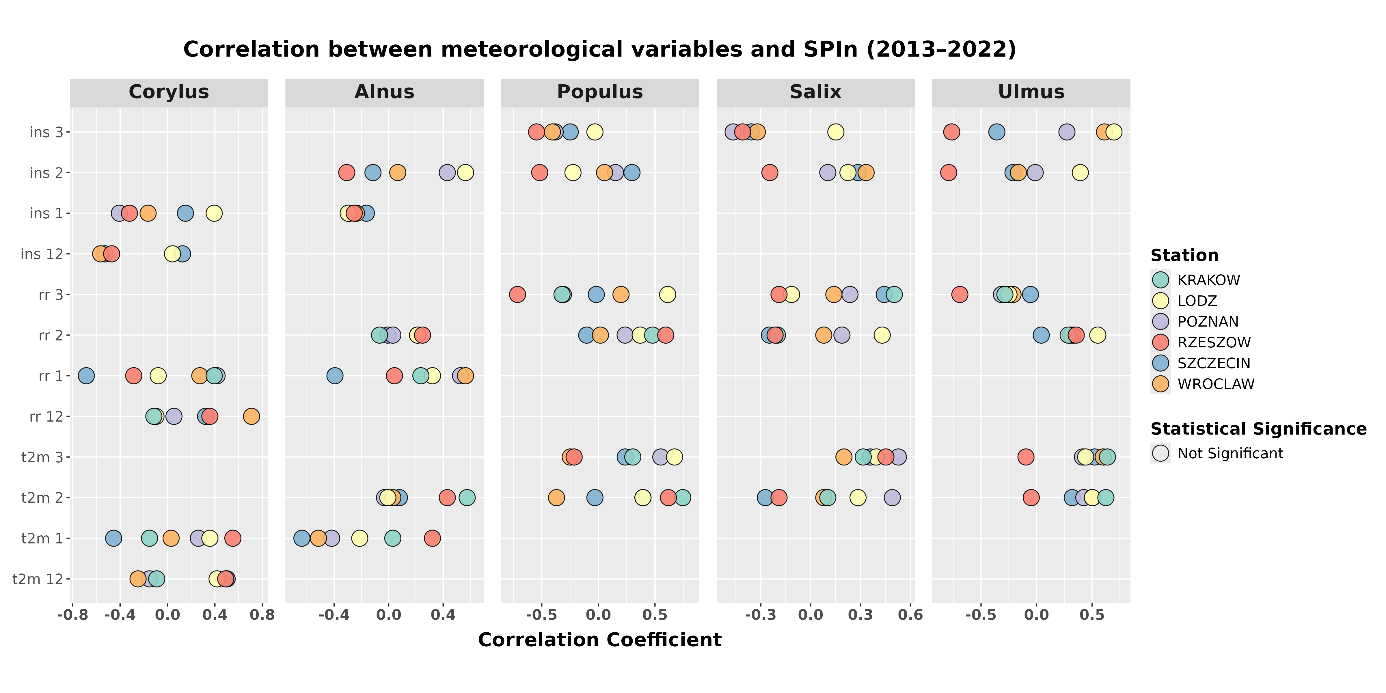


Figure 1S. Significance of the effect of variables on SPIn 2003-2012 and 2013-2022, calculated using the 95 method (statistically significant marked as * for p < 0.05, ** for p < 0.01, and *** for p < 0.001). Each variable is labeled with the corresponding month number, while the x-axis varies according to the taxon under study.


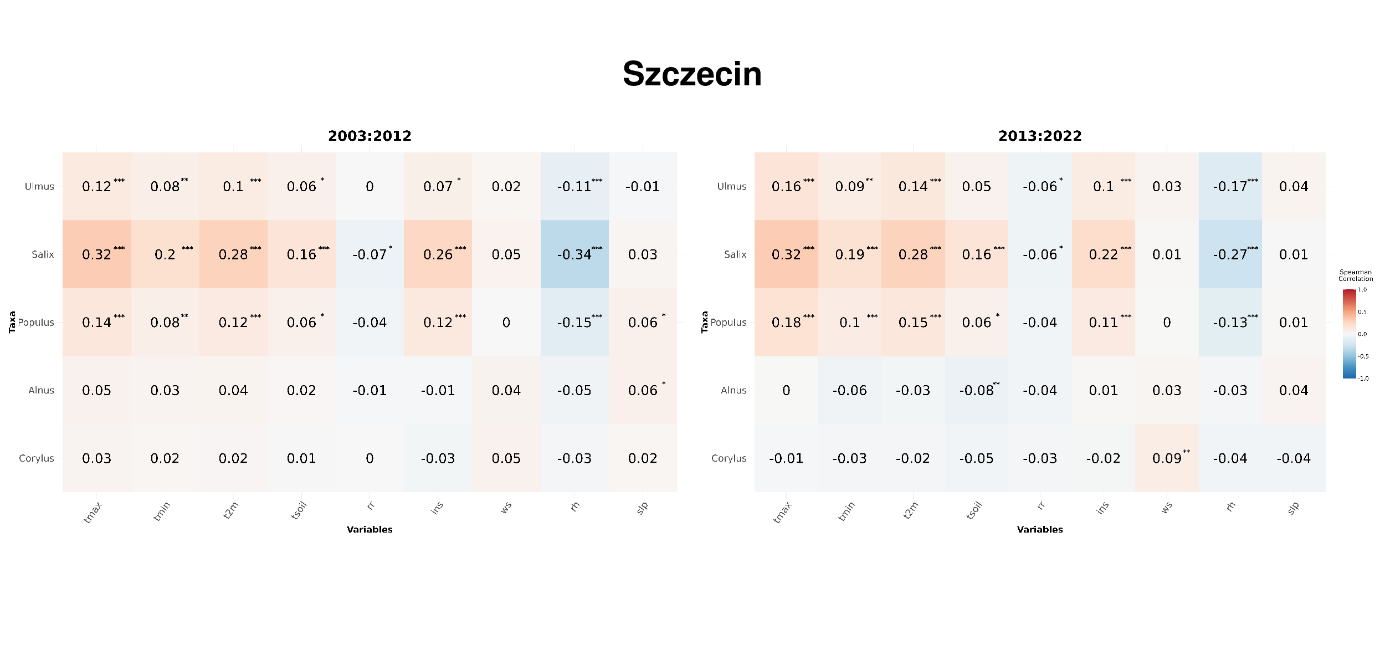


Figure 2S. Significance of the effect of variables on pollen grain concentration in Szczecin: comparison between the periods 2003–2012 and 2013–2022, calculated using the 95 method (statistically significant marked as * for p < 0.05, ** for p < 0.01, and *** for p < 0.001).


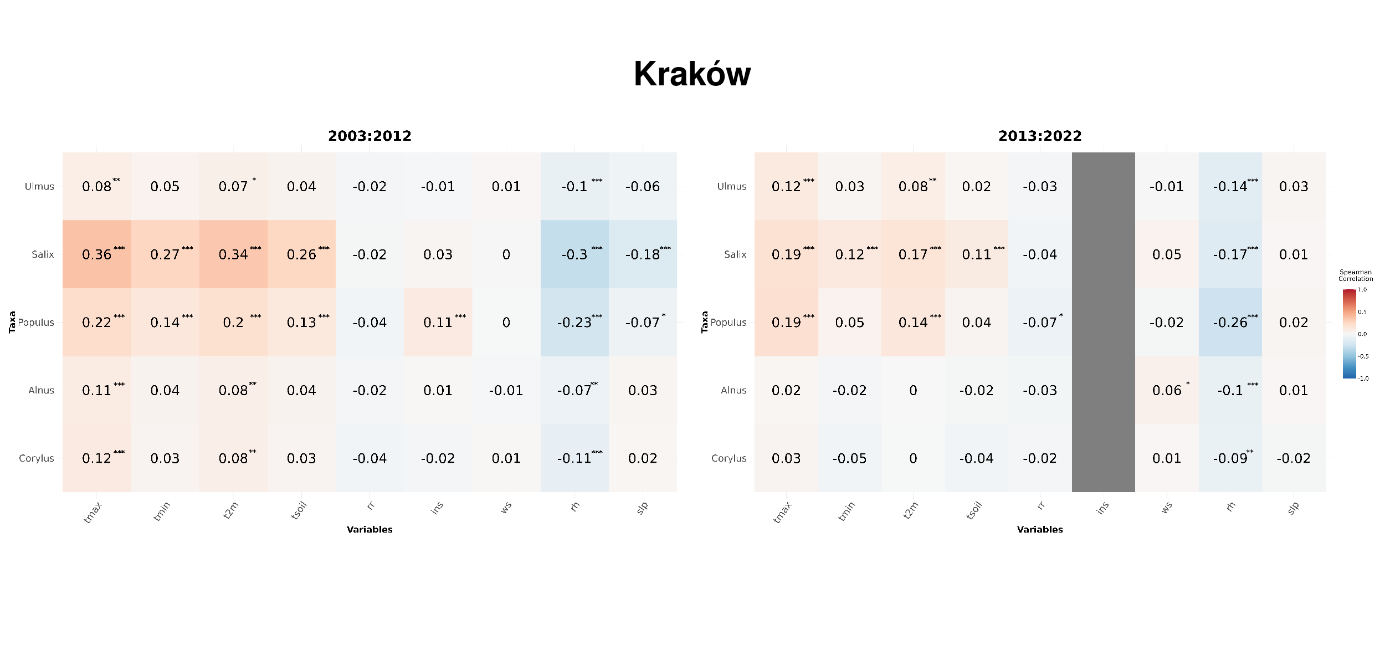


Figure 3S. Significance of the effect of variables on pollen grain concentration in Kraków: comparison between the periods 2003–2012 and 2013–2022, calculated using the 95 method (statistically significant marked as * for p < 0.05, ** for p < 0.01, and *** for p < 0.001).


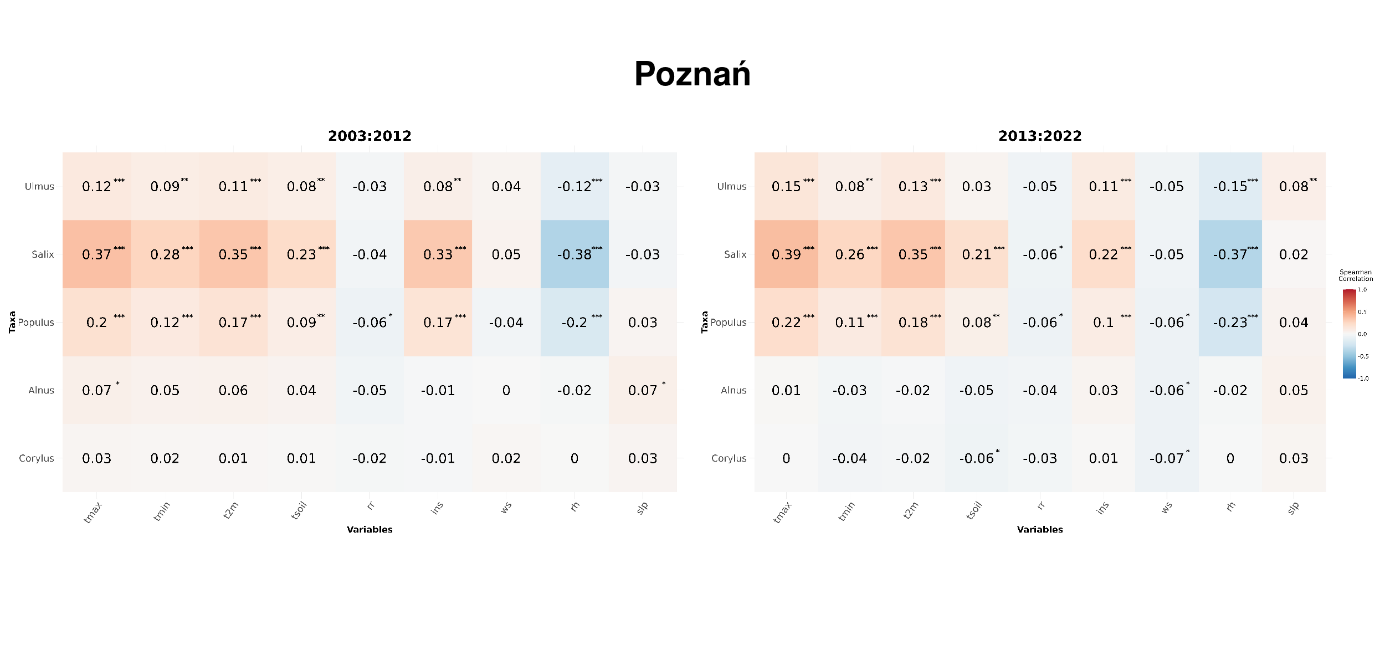


Figure 4S. Significance of the effect of variables on pollen grain concentration in Poznań: comparison between the periods 2003–2012 and 2013–2022, calculated using the 95 method (statistically significant marked as * for p < 0.05, ** for p < 0.01, and *** for p < 0.001).


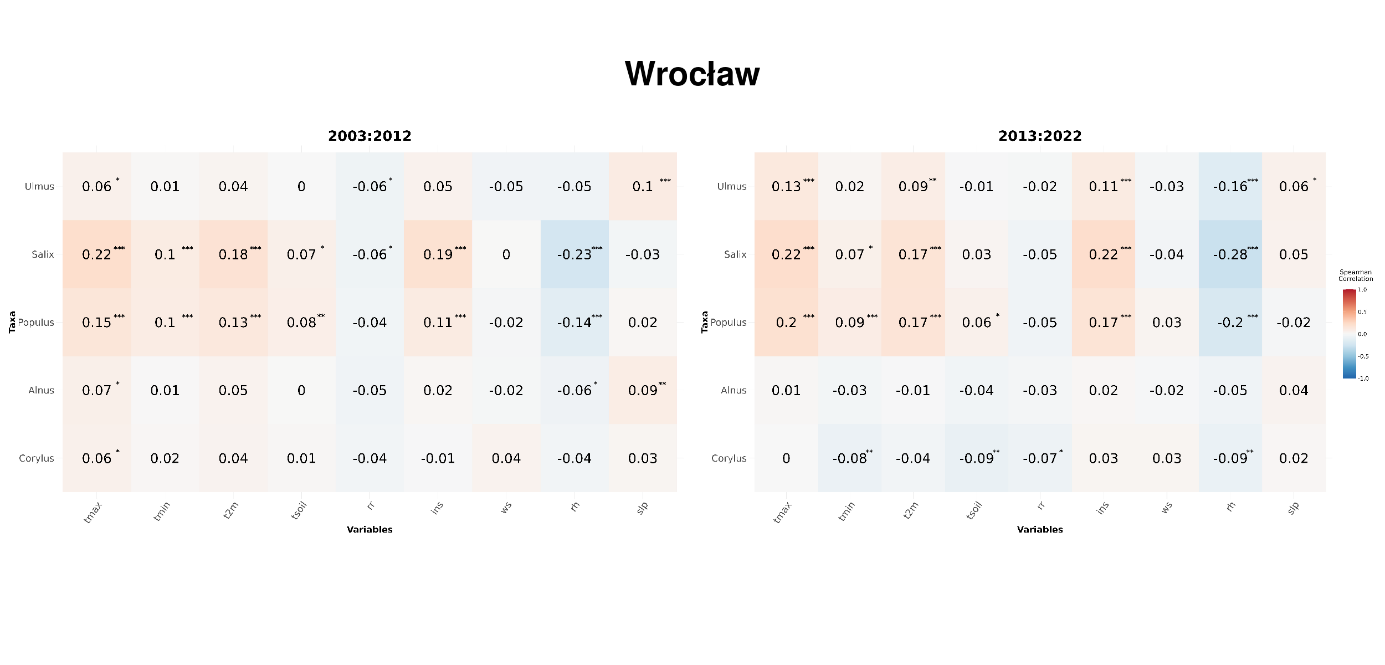


Figure 5S. Significance of the effect of variables on pollen grain concentration in Wrocław: comparison between the periods 2003–2012 and 2013–2022, calculated using the 95 method (statistically significant marked as * for p < 0.05, ** for p < 0.01, and *** for p < 0.001).


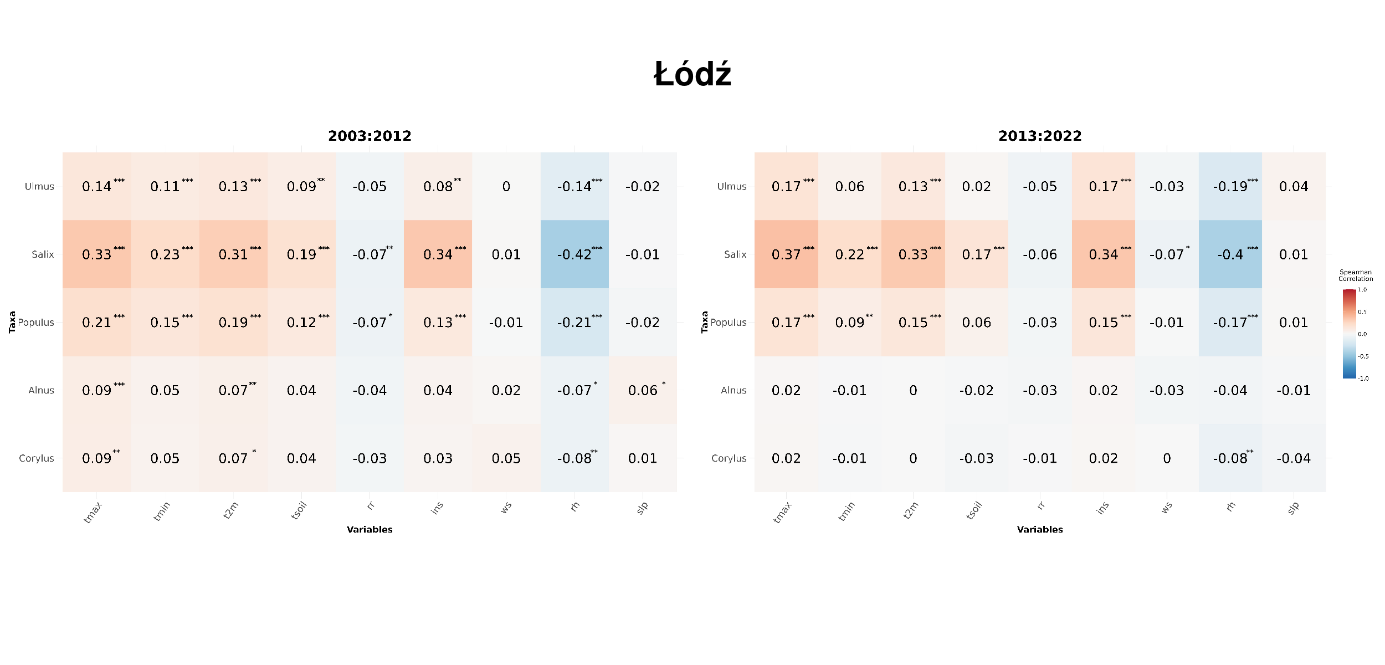


Figure 6S. Significance of the effect of variables on pollen grain concentration in Łódź: comparison between the periods 2003–2012 and 2013–2022, calculated using the 95 method (statistically significant marked as * for p < 0.05, ** for p < 0.01, and *** for p < 0.001).


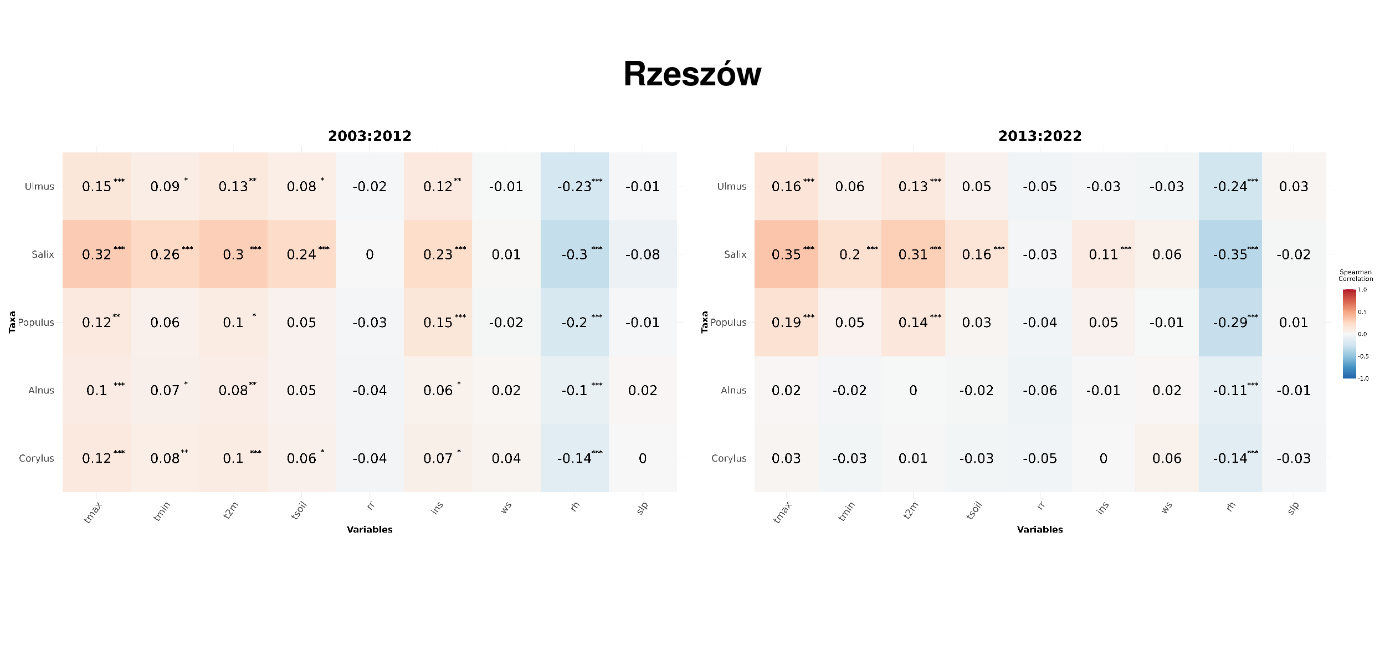
 Figure 7S. Significance of the effect of variables on pollen grain concentration in Rzeszów: comparison between the periods 2003–2012 and 2013–2022, calculated using the 95 method (statistically significant marked as * for p < 0.05, ** for p < 0.01, and *** for p < 0.001).
